# Cohesive Growth in Europe: A Tale of Two Peripheries

**DOI:** 10.1007/s10272-021-0964-y

**Published:** 2021-04-06

**Authors:** Michael Dauderstädt

**Affiliations:** Bonn, Germany

## Abstract

Over the last two decades, income disparities between EU member states tended to decline, particularly before the financial crisis. While Central and Eastern Europe caught up with the EU average, Southern Europe fell behind after 2009. Catch-up growth in both peripheries relied on nominal convergence (real appreciation) and foreign capital. Further growth can and should be fostered by an economic policy that does not neglect domestic demand, stabilises capital markets and invests in research, education, health and intangibles.

The European Union has long shown large income disparities between its member states (measured by average per capita income). This was mainly due to the entry of new member states with significantly lower income levels to the EU between 1973 and 2013. Reducing these disparities and achieving cohesion requires catch-up growth.

The convergence of income levels in Europe has been the subject of various studies[1] and is anchored in the treaties as a goal of the EU. The most important instrument to achieve it has been EU funds, but they are relatively small and amount to less than 0.5% of the EU's GDP. However, the track record of EU cohesion policy (documented in regular reports by the Commission) has been modest.[2] That leaves the question open as to whether the income disparities would be even greater without the EU's support. In this article, the development of income disparities are described, their causes examined and possible policies for reducing them are discussed.

1 Barro and Sala-i-Martin (1991); for an overview of the literature, see Dauderstädt (2014), Table 12.

2 The latest report has been the Seventh report on economic, social and territorial cohesion, European Commission (2017).

**Michael Dauderstädt**, freelance consultant and writer, Bonn, Germany.

## The size and evolution of income inequality between member states and regions

The size and development of income inequality between member states also depends on the choice of indicators. This applies to income itself (market income, disposable income, GDP) and its conversion into euros. Converted at exchange rates, the income disparities are significantly greater than those at purchasing power parity (PPP), as purchasing power is higher in poorer countries where the price level is lower (see Table 2). But the choice of indicators of inequality also influences the result. Some indicators measure relative inequality (such as quintile ratios or the coefficient of variation), some measure absolute disparities (e.g. standard deviation, Gini). A declining standard deviation indicates sigma convergence (see Figure 1) while beta convergence, i.e. stronger growth in poorer countries, improves indicators of relative inequality. Paradoxically, in the short and medium run, beta convergence does not necessarily imply sigma convergence and indicators of relative and absolute inequality may show opposite trends (Barro and Sala-i-Martin, 1991; Islam, 2003; Dauderstädt, 2020).

In certain periods of time and for certain groups of member states, beta convergence could be observed (see Table 2). At the same time there is a lack of sigma convergence, i.e. the dispersion of incomes does not decrease – except during the crisis. Figure 1 shows the development of the standard deviation between the per capita incomes of the member states and the regions at NUTS 1 and NUTS 2 levels.[3] Income disparities have increased

3 *Nomenclature des unités territoriales statistiques* (NUTS) indicates the set of regions of the EU. NUTS 1 comprises 98 regions; NUTS 2 is more disaggregated and comprises 277 regions.

Figure 1

**Standard deviation of per capita incomes in the EU28, 2000-2019**

in euros

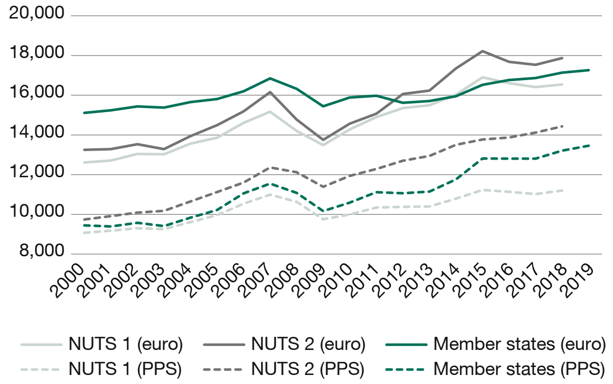

Notes: PPS stands for purchasing power standard; data for NUTS 1 and 2 are lacking for France until 2014 and generally for 2019.

Source: Eurostat, author's calculations.

Figure 2

**Coefficient of variation of per capita incomes in the EU28, 2000-2019**

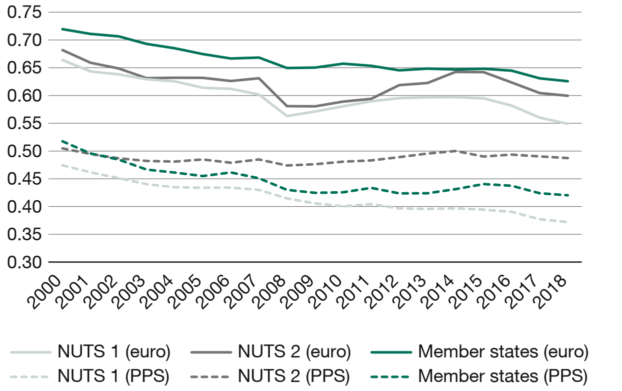

Notes: PPS stands for purchasing power standard; data for NUTS 1 and 2 are lacking for France until 2014 and generally for 2019.

Source: Eurostat, author's calculations.

almost continuously, except in the 2007-2009 period when, during the financial crisis, the incomes of the poorer countries of Central Eastern Europe (CEE) fell less sharply than those of the relatively richer member states. As mentioned above, the standard deviation for incomes measured in purchasing power standards (PPS) is lower than that for incomes calculated in euros using exchange rates. Inequality has increased most at the NUTS 2 level, which indicates growing regional disparities.

If, on the other hand, one chooses indicators of relative inequality such as the coefficient of variation (standard deviation/mean) or the ratio of the highest to the lowest income, a different picture emerges, as Figure 2 shows. An almost steady decline in the coefficient of variation can be observed for the member states and NUTS 1; but here too, the greater income disparity is evident at the NUTS 2 level. Inequality fell more sharply until 2008, after which the convergence process weakened. The value levels for income in PPS and in euros show the expected difference (lower in PPS).

If the ratio of the highest to the lowest income is selected as the indicator, a similar picture of steady decline emerges, albeit at very different levels. Looking at the NUTS 2 level, the richest sub-region in the EU was Inner London West with a per capita income of €142,100 in 2000 and the poorest was Nord-Est in Romania with €1,600. In 2018, Inner London West had increased its average per capita income by 50% to €213,400, but for the poorest region (then Severozapaden in Bulgaria), it had jumped by 225% to €5,200. This paradoxically means that the absolute gap has increased further (from €141,500 in 2000 to €208,200 in 2018), while the relative ratio declined from 109 to 41.

Another frequently used indicator of relative inequality is the quintile ratio (S80/S20 ratio), which gives the relation between the income of the richest one-fifth (quintile) of the population and that of the poorest. In Table 1, the EU population quintiles (approximately 102 million people each) were constructed using different methods. If one adds up the member states (or parts of them) to get the

Table 1

**Quintile ratios of income in the EU**

| Compared entity | Neglected disparities | 2000 | 2010 | 2018/19 |
|---|---|---|---|---|
| Member states PPS | Income disparities within member states | 3.10 | 2.02 | 1.80 |
| Member states | | 5.48 | 3.81 | 3.15 |
| NUTS 1 PPS | Income disparities within NUTS 1 regions | | | 2.48 |
| NUTS 1 | | | | 4.02 |
| NUTS 2 PPS | Income disparities within NUTS 2 regions | 4.00 | 2.80 | 2.77 |
| NUTS 2 | | | | 4.49 |
| Households PPS | Income disparities within national quintiles | | 6.99 | 5.87/5.56 |
| Households | | | 9.48 | 8.45/7.90 |
| To compare: USA states | Income disparities within federal states | | | 1.69 |

Note: Analysis of the author at a time when these data for 2000 and 2010 were still available from Eurostat.

Source: Eurostat, author's calculations.

20% of the EU population, one neglects the differences within the member states. Similarly, when constructing the quintiles from NUTS 1 or NUTS 2 regions, the disparities within the regions are neglected. The household level takes account of income differences both within and between member states, since it uses the national quintiles (but also ignores the differences within the quintiles). All ratios were calculated measuring the respective incomes at exchange rates and PPP. Table 1 shows that the inequality increases with the granularity (choice of smaller regions). However, at all levels, the inequality decreases over time, which is in line with the data presented above.

### The impact of the COVID-19 pandemic on cohesion

The coronavirus pandemic has affected the EU member states to different extents. Countries that depend on tourism suffered more than those who rely on manufacturing. Less indebted member states such as Germany could afford stronger fiscal support programmes than already highly indebted countries. These qualities, however, are not closely correlated with levels of per capita income. In a similar way, it is not clear if poorer countries will experience a stronger decline of GDP than richer member states. As Figure 3 shows, the richest countries tended to have somewhat weaker recessions in 2020, but the dispersion within both groups, poorer (left of vertical line) and richer (right of vertical line) member states, is very high.

Given this picture, it appears likely that the pandemic and the ensuing crisis did not substantially affect overall inequality between countries. But it has hit the Southern periphery much harder than the Eastern periphery and reinforced the already persistent trend visible in Table 2.

### Catch-up growth in Europe

Catch-up growth, i.e. higher growth rates in the poorer countries relative to the richer ones (beta convergence) does not reduce the absolute income gap immediately, but is a necessary condition for long-term (sigma) convergence (Islam, 2003). We consider three specific groups of (originally) poorer member states corresponding to the three waves of enlargement – Ireland, GPS (Greece, Portugal and Spain) and CEE – with different growth performances in the past and compare them with the group of high-income member states (the north-west of the EU).

As Table 2 shows, the poorer member states of CEE showed significantly higher income growth between 2000 and 2019 (99.6% and 167.9% at PPS) than the richer core of the EU (18.6% and 49% at PPS respectively), whereby the absolute income gap, measured at exchange rates, decreased only slightly and at PPS significantly. Southern European countries, on the other hand, lagged behind the richer north-west. Greece, Portugal and Spain experienced higher growth than the EU core only until 2008 with 14.9% (at PPS even 38.2%), while the core grew by 10.7% (respectively 24.1% at PPS). Ireland started its dramatic catching-up process around 1990. Its per capita income grew from 70% of

Figure 3

**The impact of the COVID-19 pandemic depending on pre-crisis per capita income**

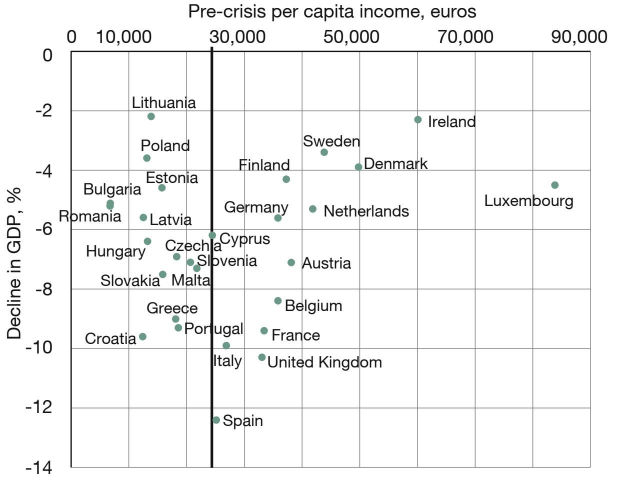

Sources: Eurostat, author's calculations.

Table 2

**Income levels and growth rates of different EU country groups, 2000-2019**

| | 2000 | 2008 | 2019 | 2000-2008 | 2008-2019 | 2000-2019 |
|---|---|---|---|---|---|---|
| | Per capita income (in euros, not PPP adjusted) | | | Growth rate (in %) | | |
| North-west | 29,133 | 32,254 | 34,550 | 10.7 | 7.1 | 18.6 |
| CEE | 6,419 | 9,634 | 12,813 | 50.1 | 33.0 | 99.6 |
| GPS | 19,900 | 22,858 | 23,079 | 14.9 | 1.0 | 16.0 |
| Ireland | 33,281 | 36,769 | 60,084 | 16.2 | 55.4 | 80.5 |
| | Per capita income (in euros, PPP adjusted) | | | Growth rate (in %) | | |
| North-west | 23,929 | 29,692 | 35,649 | 24.1 | 20.1 | 49.0 |
| CEE | 8,776 | 15,538 | 23,509 | 77.1 | 51.3 | 167.9 |
| GPS | 18,207 | 25,163 | 27,025 | 38.2 | 7.4 | 48.4 |
| Ireland | 26,455 | 34,971 | 61,990 | 32.2 | 77.3 | 134.3 |

Note: North-west refers to EU15, excluding GPS; GPS refers to Greece, Portugal, Spain; CEE refers to Bulgaria, Czechia, Estonia, Hungary, Latvia, Lithuania, Poland, Romania, Slovakia and Slovenia.

Source: Eurostat, author's calculations.

Figure 4

**Catch-up processes of Eastern and Southern Europe**

per capita income in euros and PPS of the catching-up region as a percentage of the per capita income of the north-west of the EU

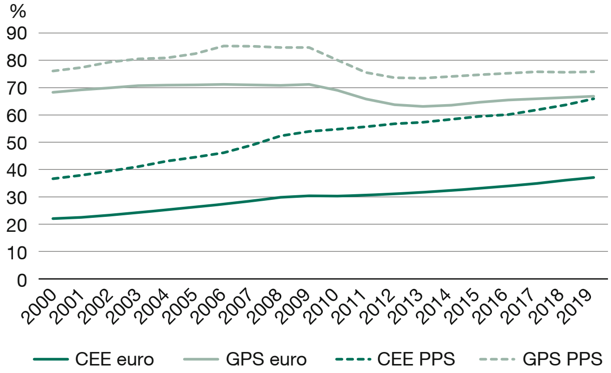

Notes: North-west refers to EU15, excluding GPS; GPS refers to Greece, Portugal, Spain; CEE refers to Bulgaria, Czechia, Estonia, Hungary, Latvia, Lithuania, Poland, Romania, Slovakia and Slovenia.

Sources: Eurostat, author's calculations.

Figure 5

**Net international investment position of Eastern and Southern Europe**

in % of GDP

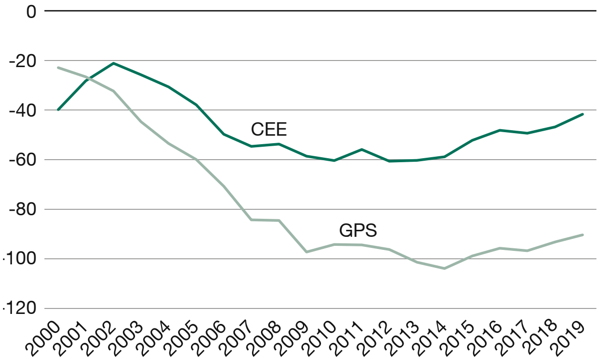

Notes: CEE refers to Bulgaria, Czechia, Estonia, Hungary, Latvia, Lithuania, Poland, Romania, Slovakia and Slovenia; GPS refers to Greece, Portugal and Spain.

Sources: Eurostat, author's calculations.

the EU15 average to around 120% in 10 years and, despite already being a high-income country, continued to grow significantly faster than the other richer member states after 2000.

Figure 4 shows the continuous catching-up process of the new member states of CEE, albeit faster before the financial crisis of 2009 than afterwards, while Greece, Portugal and Spain fell relatively behind after 2009. One also can observe the familiar pattern of relatively higher income levels when measured at PPS.

## Different patterns of catch-up growth

As Table 2 shows, different parts of the lower-income EU periphery performed differently due to different initial conditions and policies. This is demonstrated by the following brief histories.

Ireland, the most successful poorer entrant, has used tax dumping and the resulting transfer pricing to attract massive foreign direct investments, which added fictitious value to its GDP. One medium-term consequence was that, although GDP rose sharply, gross national income lagged behind and the wage share plummeted (Dauderstädt, 2001). In the longer term, however, there were further demand impulses and rising price levels (from 92% of the EU average in 1995 to 119.5% in 2008), which were corrected briefly in the financial crisis, but ultimately underpinned the catching-up success.

Greece, Spain and Portugal had lower growth rates. Greece in particular started to fall behind relative to the core member states already in 1981 (after joining the European Community), while Spain and Portugal slowly caught up from 1986 onwards (also thanks to more favourable world market conditions). After 2000, all three countries benefitted from the euro (e.g. lower real interest rates). Since their growth was higher than in the EU core, they attracted capital inflows from there, which helped finance the credit expansion in the South (see Figure 5). Current account deficits accumulated (see Figure 6), and prices and wages rose faster than in the core. The financial crisis brought the catch-up process financed in this way to a sudden stop. Counterproductive policies of the EU in the face of the sovereign debt panic in 2010 and the austerity programmes that were subsequently imposed produced deep recessions without solving the debt problems (Dauderstädt, 2016).

CEE performed much better than the South. It partially followed the Irish path by attracting parts of international value chains (especially from German industry), but never achieved the attractiveness of Ireland nor the volume of investment per capita. The strong migration from Eastern Europe to the Western EU created income (remittances) and increased per capita income, but also deprived the economies of manpower. But for Eastern Europe, too, as in the South, higher inflation and foreign debt were part of the catching-up process: the unweighted average price level rose from 48.6% of the EU average in 2000 to 67% in 2019. The current account deficits increased between 2000 and 2008 and the net foreign position deteriorated by 40 percentage points of GDP (see Figures 6 and 7). However, CEE benefitted from huge transfers from EU funds (1%-2% of GDP per year). The financial crisis slowed down the catching-up process in CEE, too (see Table 2).

Figure 6

**Current account balance of Eastern and Southern Europe, 2000-2019**

in % of GDP

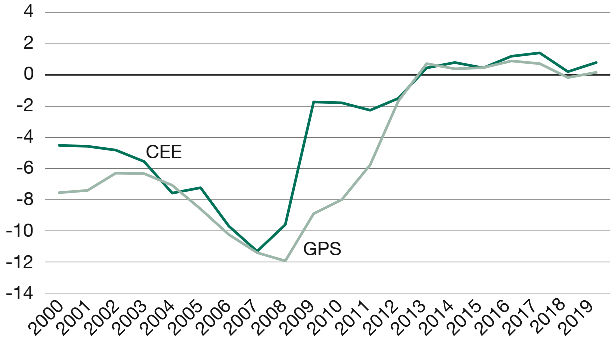

Notes: CEE refers to Bulgaria, Czechia, Estonia, Hungary, Latvia, Lithuania, Poland, Romania, Slovakia and Slovenia; GPS refers to Greece, Portugal and Spain.

Sources: Eurostat, author's calculations.

Figure 7

**Price level in Eastern and Southern Europe, 2000-2019**

index, EU28 = 100

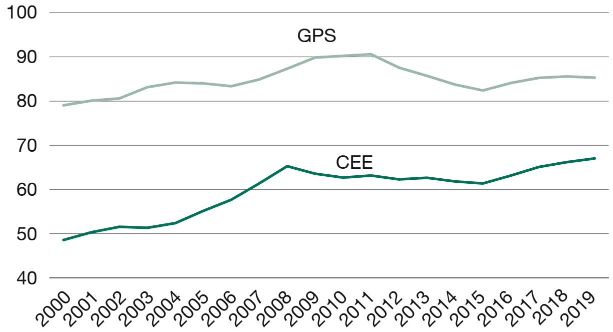

Notes: CEE refers to Bulgaria, Czechia, Estonia, Hungary, Latvia, Lithuania, Poland, Romania, Slovakia and Slovenia; GPS refers to Greece, Portugal and Spain.

Sources: Eurostat, author's calculations.

## Nominal and real convergence

Catch-up growth has two components: nominal and real convergence. Nominal convergence reflects the rise of prices and incomes in nominal terms. A country with, say, half the EU's average income per capita could virtually achieve parity by doubling all prices and incomes, albeit with problematic consequences such as a surge of imports, decline of exports, bankruptcies of exposed firms and rising unemployment. A real case of rapid nominal growth has been East Germany when it adopted the Deutschmark at an overvalued exchange rate. It could only survive the consequences thanks to massive transfers from West Germany.

True real convergence must be based on a rise of production and value added that depends on increasing productivity and hours worked. Poorer member countries are poorer because their labour productivity is lower while their workers often work more hours (sometimes more than 2,000 per year) than those in richer member states (often with less than 1,500). However, less employment (lower participation rates, higher unemployment) can and often does reduce the impact of more hours worked per employee. Structural change (like shifting labour from the agriculture to the manufacturing industry and trade-induced specialisation), investment in physical, human and intangible capital, and the adoption of better technologies and management techniques increase the average productivity of a country's economy.

Growing levels of employment and productivity depend not only on supply-side factors but on increasing demand, too. Real productivity (measuring real output) increases when capacities are fully utilised and economies of scale occur. Higher demand also tends to raise output prices, which increases nominal or monetary productivity and, in the end, GDP. That demand does not have to be foreign demand for exports. Economies can grow by expanding their domestic market and the production of non-tradable goods and services. Indeed, this is the only way the world economy is growing.

However, successful real convergence implies nominal convergence, too. While in catching-up economies, some industries approach the productivity frontier defined by the most developed economies, real productivity in other sectors such as services (e.g. education, health care, public administration, music industry) will remain the same. Incomes in these industries must rise by increasing (relative) prices, which, in turn, increases the nominal productivity (monetary value added per worker or hour) in these industries. In economic theory, this process is known as the Balassa–Samuelson effect. It implies a real appreciation of the catching-up country's currency via higher inflation and/or nominal revaluation of the exchange rate. Actually, prices in the periphery did approach average EU levels, as seen in Figure 7. The rise was faster before the financial crisis and slowed down afterwards including a temporary reversal that was stronger in Greece, Portugal and Spain due to the austerity enforced there.

While exchange rates were fixed for euro area members Greece, Portugal and Spain, most CEE countries could have used currency appreciation and devaluation to manage nominal convergence. They used this option modestly, probably because they were constrained by the wish to join the euro area and/or they did not want

Figure 8
**NEER and REER in Eastern and Southern Europe**
unweighted average, 2010 = 100

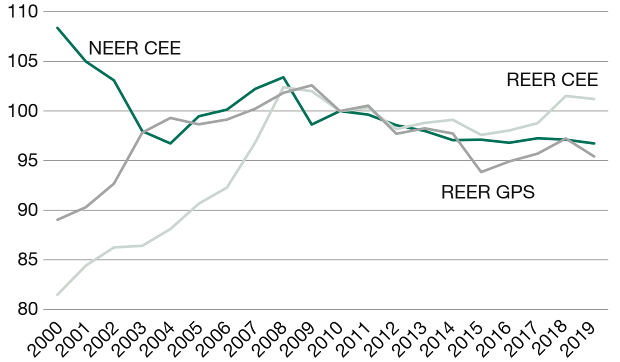

Notes: CEE refers to Bulgaria, Czechia, Estonia, Hungary, Latvia, Lithuania, Poland, Romania, Slovakia and Slovenia; GPS refers to Greece, Portugal and Spain.

Sources: Eurostat, author's calculations.

to increase their real foreign debt burden. Until 2004, nominal devaluation dominated but has been overcompensated by inflation leading to an appreciation of the real effective exchange rate (REER) as can be seen in Figure 8. After 2004, the nominal effective exchange rate (NEER) appreciated in CEE, supporting the further rise of the REER. After the financial crisis, the decline of the average NEER was driven by devaluations of the Romanian Lei, the Polish Zloty and the Hungarian Forint while other countries had adopted the euro or maintained a relatively fixed exchange rate to the euro (Bulgaria and Czechia). The Southern periphery had to adopt policies of internal devaluation.

The real/internal devaluations were considered necessary to rebalance the current account and to prevent a further decline of the net international investment position. Many observers had blamed the current account deficits (see Figure 6) on the higher nominal unit labour costs of Greece, Portugal and Spain, which were interpreted as declining competitiveness. In fact, between 2000 and 2008 all three countries experienced stronger export growth than the so-called "export champion" Germany. Their export market shares hardly changed between 2000 and 2008 (Kang and Shambaugh, 2016). Therefore, competitiveness did not decline if one understands it as the capacity to sell abroad. The exclusive focus on unit labour costs neglects the much more important role of financial flows, which fuelled growth in Southern Europe (Gabrisch, 2017; Storm and Naastepad, 2014). Trade balances deteriorated as imports rose in the wake of strong growth, not because exports weakened.

## Policies for cohesive growth

The EU has achieved higher convergence rates than many other integration areas or nation states (Dauderstädt, 2014). But neither market integration nor regional policy are ways to guarantee catching-up success, as Southern Italy, Eastern Germany and parts of the EU show. The different growth paths and results (see above) indicate that success depends not only on the European framework and policies, but also on national measures. Thus, we consider both the European and the national level whereby it should be borne in mind that EU membership (and even more so that of the euro area) restricts the choice of national policies.

The European internal market guarantees access for catching-up countries to the affluent markets of the richer countries, which has favoured the relocation of production to the poorer countries. But it also exposes the producers of the less developed countries to the competitive pressure of the more developed member states. The EU forbids subsidies and industrial policies like those used by the successful East Asian "tiger economies", although it tolerates subsidy-like cost reductions for companies through low wages and taxes or even demands it within the framework of austerity programmes.

Where it intervenes directly, the EU should avoid harming growth. The conditions of the Troika for the South were characterised among other things by incompatible goals (budget consolidation, current account improvement/internal devaluation, growth). Internal deflation and wage cuts lowered tax revenues and made budget consolidation more difficult. The latter, in turn, dampened growth and the austerity policy weakened spending on research, education and infrastructure, which are important for structural competitiveness. As a result, the debt ratio rose and there was hardly any improvement in competitiveness. Current accounts improved primarily because imports declined in the wake of the recession.

In general, the EU's economic policy is supply- and stability-oriented while the demand side often remains neglected. This is evident in its monetary policy and fiscal policy, and in its recommendations for national economic policy in the context of the macroeconomic imbalance procedure. Uniform targets like the 3% deficit and the 60% debt level limits for all countries make no sense because, according to the Domar growth model, these values are only compatible with a nominal growth rate of 5%. The EU should actually aim at such a nominal growth rate and tolerate moderate inflation rates of 2%-6%, in particular in poorer member states. In such a macroeconomic context, debts, which are important drivers of growth, are sustainable. With regard to member states (and candidate countries) that have not yet joined the euro area, the EU should not insist on stable exchange rates and low inflation as conditions for entry as this impedes real appreciation and catch-up growth.

The European Central Bank (ECB) and the EU should guarantee the sovereign debts of all member states of the eurozone, as the ECB has been implicitly doing, albeit belatedly since 2012, in order to relieve the pressure of the capital markets and prevent doom loops of sovereign debt panic and banking crises. As mentioned above, the successful catch-up growth before 2008 was driven by capital inflows, which need to be stabilised and immunised against sudden changes of market sentiment.

The EU funds have obviously not promoted convergence very effectively in the past, as seen in the cases of Mezzogiorno, Greece or Ireland up to 1990. East Germany also shows that such massive transfers lead to only limited success in catching up. On the other hand, it is striking that the successful CEE countries received high inflows of funds from the EU budget – between 1% and 4% of their GDP. But these funds could and should be used in a more targeted manner.

Beyond a more expansive macro policy, the EU could and should pursue a proactive industrial policy that supports the upgrading of the production and export structure of the catching-up countries through investments in education and research as well as public procurement programmes. But import substitution can also help. The EU could promote the development of alternative energy production – especially in the South – in order to both reduce energy imports and support climate protection. The coronavirus crisis has shown that the structure of international supply chains should not only be left to market forces and geopolitical capers. Relocating systemically relevant productions (e.g. medicines) from third countries to the EU would be an opportunity to support previously disadvantaged locations.

The success of growth also depends on how the countries use the international environment and European aid. Ireland did it very skillfully (albeit at the expense of others); Greece obviously less so. The prevailing opinion, which shaped European economic policy, saw supply-oriented "reforms" as the way to more growth. These consisted primarily of the liberalisation of markets, especially the labour market, of privatisation and deregulation. These measures should stimulate investment (including foreign investment) and exports. The less influential counterposition focused on strengthening domestic demand through a productivity-oriented wage policy, a strong welfare state (less inequality, at least in terms of disposable income) and an expansion of the non-tradable sector.

Governments should act on both, the supply and the demand side. The supply side is about improving the supply of factors (capital stock, infrastructure, education and upbringing) and increasing productivity. This increasingly includes investments in intangibles (brands, patents, market information, etc.), which up until now have mainly been concentrated in the highly developed countries. The demand side can be secured by appropriate fiscal policies, a well supervised credit expansion and the growth of the non-tradable sector, including housing.

It should be noted that the modern economy is no longer dominated by industrial mass production alone, but increasingly by value creation that results from the appreciation by client groups who are interested in social distinction and unique consumer opportunities (Reckwitz, 2019; Boltanski and Esquerre, 2019). In Southern Europe in particular, tourism in its various forms (mass and package tourism, individual and quality tourism, long-term stays in holiday homes) plays an important role. Here, the climate, landscape and cultural heritage can be marketed, whose value (and thus price and sales) can be improved through targeted strategies (marketing, events, the location of attractions such as museums, provision of roads and hiking trails). New approaches will be needed to counteract the dire effects of the COVID-19 crisis on the EU's Southern periphery in particular.
